# CAPTURE of the Human U2 snRNA Genes Expands the Repertoire of Associated Factors

**DOI:** 10.3390/biom12050704

**Published:** 2022-05-14

**Authors:** Joana Guiro, Mathias Fagbemi, Michael Tellier, Justyna Zaborowska, Stephanie Barker, Marjorie Fournier, Shona Murphy

**Affiliations:** 1 Sir William Dunn School of Pathology, University of Oxford, Oxford OX1 3RE, UK; guiro.joana@gmail.com (J.G.); mathias.fagbemi@path.ox.ac.uk (M.F.); michael.tellier@path.ox.ac.uk (M.T.); jwzaborowska@gmail.com (J.Z.); stephanie.barker@path.ox.ac.uk (S.B.); 2 Advanced Proteomics Facility, Department of Biochemistry, University of Oxford, South Parks Road, Oxford OX1 3QU, UK; marjorie.fournier@bioch.ox.ac.uk

**Keywords:** CAPTURE, U2 snRNA gene, transcription, SPT6, CDK12, RNA processing, polyadenylation

## Abstract

In order to identify factors involved in transcription of human snRNA genes and 3′ end processing of the transcripts, we have carried out CRISPR affinity purification in situ of regulatory elements (CAPTURE), which is deadCas9-mediated pull-down, of the tandemly repeated U2 snRNA genes in human cells. CAPTURE enriched many factors expected to be associated with these human snRNA genes including RNA polymerase II (pol II), Cyclin-Dependent Kinase 7 (CDK7), Negative Elongation Factor (NELF), Suppressor of Ty 5 (SPT5), Mediator 23 (MED23) and several subunits of the Integrator Complex. Suppressor of Ty 6 (SPT6); Cyclin K, the partner of Cyclin-Dependent Kinase 12 (CDK12) and Cyclin-Dependent Kinase 13 (CDK13); and SWI/SNF chromatin remodelling complex-associated SWI/SNF-related, Matrix-associated, Regulator of Chromatin (SMRC) factors were also enriched. Several polyadenylation factors, including Cleavage and Polyadenylation Specificity Factor 1 (CPSF1), Cleavage Stimulation Factors 1 and 2 (CSTF1,and CSTF2) were enriched by U2 gene CAPTURE. We have already shown by chromatin immunoprecipitation (ChIP) that CSTF2—and Pcf11 and Ssu72, which are also polyadenylation factors—are associated with the human U1 and U2 genes. ChIP-seq and ChIP-qPCR confirm the association of SPT6, Cyclin K, and CDK12 with the U2 genes. In addition, knockdown of SPT6 causes loss of subunit 3 of the Integrator Complex (INTS3) from the U2 genes, indicating a functional role in snRNA gene expression. CAPTURE has therefore expanded the repertoire of transcription and RNA processing factors associated with these genes and helped to identify a functional role for SPT6.

## 1. Introduction

Human small non-coding RNAs (snRNAs) are required for expression of the vast majority of our protein-coding genes due to their important roles in pre-mRNA processing [1,2]. Unlike the majority of mRNAs, the mammalian pol II-dependent snRNAs are not spliced or polyadenylated and 3′ end formation is directed by a gene-specific 3′ box rather than a poly(A) site [2,3,4,5]. Cleavage of nascent transcripts by the Integrator Complex [6,7,8] just upstream of the 3′ box produces pre-snRNAs that are further processed by cap hypermethylation, 3′ trimming, and association with proteins to make mature snRNPs [1,5]. These snRNA genes have a specialized promoter comprising an enhancer-like distal sequence element (DSE) and an essential proximal sequence element (PSE) that functions as the core promoter [2,3,4,5].

Many proteins involved in transcription of the pol II-dependent human snRNA genes and processing of the transcripts have been identified. These include Oct-1 that binds directly to the DSE [9,10,11] and PSE Transcription Factor (PTF)/PSE-Binding Protein (PBP)/snRNA-Activating Protein complex (SNAPc), a multisubunit factor that binds to the PSE [9,10,12,13,14,15]. Oct-1 helps to recruit PTF to the promoter [9,10] to nucleate a pre-initiation complex (PIC) comprising general transcription factors TFIIA, B, C, E, F, and H; TBP; and some TBP-associated factors (TAFs) [16], all factors also involved in initiation of transcription of protein-coding genes. However, the TBP–TAF complex on the U2 snRNA genes—which we have termed the snTAFc—has a complement of TAFs, which is different to canonical TFIID as TAF7 is missing [17], although TAF7 is found on the U1, U4, U5, and U11 snRNA genes [18]. Subunits of Mediator, a large 26-subunit complex [19,20] have also been found associated with pol II-transcribed snRNA genes [18,21]. Mediator is recruited to the pre-initiation complex (PIC) of protein-coding genes and acts as a binding platform for interaction between the transcription factors bound to sequences in protein-coding gene promoters and pol II [19,20].

In addition, a specialized little elongation complex (LEC) is recruited to these genes [21,22] to facilitate initiation and elongation and cleavage upstream of the 3′ box is carried out by the INTS11 subunit of the Integrator Complex, which is a CPSF73 homologue [6,8]. The Med26 subunit of the Mediator complex helps to recruit the LEC [18,21], which in turn helps to recruit Integrator [21,23].

The negative elongation factor (NELF) and DRB sensitivity-inducing factor (DSIF), comprising SPT4 and SPT5, play key roles in regulating the early elongation checkpoint during transcription of protein coding genes [24]. NELF and DSIF also interact with Integrator to facilitate 3′ box dependent processing [25] and NELF co-operates with CTCF to ensure termination of transcription of snRNA genes [26,27].

Phosphorylation of residues in the Tyr1Ser2Pro3Thr4Ser5Pro6Ser7 repeats of the carboxyl-terminal domain (CTD) of the large subunit of pol II also play a role in expression of human snRNA genes [2]. Inhibition of the cyclin-dependent kinase (CDK)9 subunit of positive transcription elongation factor b (P-TEFb), which is a CTD Ser2 kinase, causes failure to recognise the 3′ box [28,29]. Mutation of Ser7 of the CTD also affects transcription of the human U2 genes in addition to disrupting Integrator recruitment and consequently affecting 3′ box-directed RNA 3′ end processing [30]. As Integrator has been shown to recognise a double Ser2P/Ser7P phosphomark [31], inhibiting CDK9 would also disrupt Integrator recruitment. Ser7P is instead dependent on the CDK7 subunit of TFIIH [32,33] and TFIIH is associated with the U2 snRNA genes in vivo [32]. The CTD Ser5P phosphatase RPAP2 also helps to recruit the Integrator Complex to snRNA genes [34].

More than 30 years ago now, it was shown that the 3′ box is only efficiently recognized if transcription is initiated from a pol II-dependent snRNA gene promoter [35,36]. Many of the transcription factors involved in transcription of the snRNA genes are shared with protein-coding genes, including the Integrator Complex [8]. PTF and the LEC stand out as snRNA gene-type specific. However, the molecular mechanism of the link between the promoter and the 3′ box remains elusive.

In order to obtain a more complete picture of the proteins associated with the pol II-dependent snRNA genes, we have used the CAPTURE system, where biotinylated, enzymatically dead (d) Cas9 is directed to genes by guide RNAs in order to pull-down the gene region with associated proteins after crosslinking [37]. We have chosen the U2 snRNAs genes as they are tandemly repeated approximately 15 times in the haploid human genome [38,39]. As the U2 locus is likely to be triploid in HEK293 cells [40], we have used these cells as our experimental system. We have shown that dCas9 is specifically directed to the U2 gene locus with guides targeting the DSE, PSE, and 3′ box. In addition, pol II association with these genes was not affected by dCas9 targeting.

Using the CAPTURE system targeting the DSE or PSE, many factors already shown or expected to be associated with human snRNA genes are specifically enriched, including, pol II, CDK7, NELF, the SPT5 subunit of DSIF, MED23, TFIIS, the SSRP1 subunit of the FACT complex [26], and several subunits of the Integrator Complex. The histone chaperone and elongation factor SPT6 [41,42,43,44], Cyclin K, the partner of CD12 and CDK13 [45], and the SWI/SNF chromatin remodeller-associated SMARC factors [46] were also enriched. In addition, dCas9-mediated pull-down enriched the polyadenylation factors CSTF2, CSTF1, and CPSF1. We have previously shown that CSTF2 and the other polyadenylation factors, Pcf11 and Ssu72, are associated with the human U1 and U2 genes and that Pcf11 and Ssu72 function primarily as transcription terminators rather than RNA 3′-end processing factors [47].

ChIP-seq and ChIP-qPCR of the U2 genes validates the association of SPT6 and CDK12 with these genes. In addition, knockdown of SPT6 causes loss of subunit 3 (INTS3) of the Integrator Complex, indicating that SPT6 helps to recruit Integrator to cleave the nascent RNA upstream of the 3′ box.

CAPTURE has therefore expanded the repertoire of transcription and RNA processing factors associated with the U2 genes, helped to identify SPT6 as a key player in Integrator recruitment to these genes, and suggested a role for the kinase CDK12 in their expression.

## 2. Materials and Methods

### 2.1. HEK293 Cell Culture

Human embryonic kidney (HEK) 293 were grown in Dulbecco’s modified Eagle’s medium (DMEM) (Sigma-Aldrich, Gillingham, UK) with 10% fetal bovine serum, 2 mM l-glutamine, 50 U/mL penicillin, and 50 µg/mL streptomycin and incubated at 37 °C in 5% CO_2_.

### 2.2. dCas9 and Guide Cloning and Guide Design

The mutation H840A was introduced into Cas9 by PCR of the FCas9 nickase (D10A) gene in pX462 (pSpCas9n(BB)-2A-Puro (PX462) V2.0 was a gift from Feng Zhang (Addgene plasmid no. 62987; http://n2t.net/addgene:62987 (accessed on 1 March 2022); RRID:Addgene_62987)) and cloning back into the pX462 backbone [48]. Guides were cloned into this backbone using *Bbs*I. The dCas9BAP and BirA plasmids are described in [37] and are available from Addgene (pEF1a-FB-dCas9-puro and pEF1a-BirA-V5-neo). For cloning of the guides for ectopic expression with dCas9BAP, the Cas9 was removed from pX462 by digestion and ligation before cloning the guides. Guides RNAs (Table 1) were designed using the online Guide Resources Tool developed by the Zhang la [48].

### 2.3. Stable Cell Line Production

The vectors encoding dCas9BAP and BirA were linearized and transfected into HEK293 cells and the cells were selected by puromycin (10 μg/mL) and maintained in 1 μg/mL puromycin (Sigma-Aldrich, Gillingham, UK)).

### 2.4. CAPTURE and Proteomics

CAPTURE was carried out essentially as described in [37]. As a control, 4 × 10⁶ to 1 × 10⁷ dCas9BAP/BirA HEK293 stable cells were untransfected (dCas9 alone), or they were transiently transfected with a sequence-specific DSE or PSE guide RNA for 24 h, followed by cross-linking with 2% formaldehyde for 10 min and then quenching with 0.25 M glycine for 5 min (Sigma-Aldrich, Gillingham, UK)). Cells were washed twice with PBS, scraped and lysed with 10 mL of cell lysis buffer (25 mM Tris-HCl, 85 mM KCl, 0.1% Triton X-100, pH 7.4, freshly added 1 mM DTT and complete EDTA-free protease inhibitor cocktail (Sigma-Aldrich, Gillingham, UK)) + PhosSTOP (Sigma-Aldrich, Gillingham, UK))) and rotated for 15 min at 4 °C. Cell lysates were centrifuged at 2300× *g* for 5 min at 4 °C to isolate the nuclei. The nuclei were resuspended in 5 mL nuclear lysis buffer (50 mM Tris-HCl, 10 mM EDTA, 4% SDS, pH 7.4, freshly added 1 mM DTT and complete EDTA-free protease inhibitor cocktail (Roche, Basel, Switzerland) + PhosSTOP (Sigma-Aldrich, Gillingham, UK))) and incubated for 10 min at room temperature. The suspended nuclei were then mixed with 15 mL of 8 M urea buffer and centrifuged (Sigma-Aldrich, Gillingham, UK)) at 16,100× *g* for 25 min at room temperature. Nuclei were then re-suspended in 5 mL nuclear lysis buffer and mixed with 15 mL of 8 M urea buffer (10 mM Tris, 1 mM EDTA, 8 M Urea, pH 7.4 + complete EDTA-free protease inhibitor cocktail (Roche) + PhosSTOP (Sigma-Aldrich, Gillingham, UK) and centrifuged at 16,100× *g* for 25 min at room temperature. The samples were washed twice more in 5 mL nuclear lysis buffer and mixed with 15 mL of 8 M urea buffer, followed by centrifugation at 16,100× *g* for 5 min at room temperature. Pelleted chromatin was then washed twice with 5 mL cell lysis buffer. The chromatin pellet was resuspended in 5 mL of IP binding buffer without NaCl (20 mM Tris-HCl, 1 mM EDTA, 0.1% NP-40, pH 7.5, freshly added complete EDTA-free protease inhibitor cocktail (Roche) and PhosSTOP (Sigma-Aldrich, Gillingham, UK))) and aliquoted into Eppendorf tubes. Chromatin was then subjected to sonication to 200 bp on the Q Sonica Thermocube Q800R (Q Sonica, Newtown, CT, USA) (35% amplitude, 30 s on and 30 s off for 1 h. (Fragmented chromatin was centrifuged at 16,100× *g* for 25 min at 4 °C. Supernatant was combined and NaCl was added to a final concentration of 150 mM NaCl. To prepare the streptavidin beads for affinity purification, 120 μL of streptavidin agarose slurry (Sigma-Aldrich, Gillingham, UK) was washed 3 times in 1 mL of IP binding buffer and added to soluble chromatin. After overnight incubation at 4 °C, streptavidin beads were collected by centrifugation at 800× *g* for 3 min at 4 °C. The beads were then washed 5 times with 1 mL of IP binding buffer (20 mM Tris-HCl, 1 mM EDTA, 0.1% NP-40, 150–300 mM NaCl, pH 7.5, freshly added proteinase inhibitor) and proteins were then de-crosslinked by increasing the NaCl (Sigma-Aldrich, Gillingham, UK) concentration to 3 M and boiling it at 95 °C for 1 h. The samples were sent to the Advanced Proteomics Facility, Department of Biochemistry Oxford. The samples were digested with trypsin FASP. Peptides were separated by nano liquid chromatography (Easy-nLC 1000, Thermo Fischer Scientific, Waltham, MA, USA) coupled in line a Q Exactive mass spectrometer equipped with an EASY-spray source (Thermo Fischer Scientific, Waltham, MA, USA Peptides were trapped onto a C18 PepMac100 precolumn (300 µm i.d. × 5 mm, 100 Å, Thermo Fischer Scientific, Waltham, MA, USA) using Solvent A (0.1% Formic acid, HPLC grade water). Peptides were trapped onto a C18 PepMac100 precolumn (300 µm i.d. × 5 mm, 100 Å, Thermo Fischer Scientific) using Solvent A (0.1% Formic acid, HPLC grade water). The peptides were further separated onto an EASY-spray RSLC C18 column (75 μm i.d., 50 cm length, Thermo Fischer Scientific, Waltham, MA, USA) using a 60 min linear gradient (15% to 35% solvent B (0.1% formic acid in acetonitrile)) at a flow rate 200 nL/min (Thermo Fischer Scientific, Waltham, MA, USA). The raw data were acquired on the mass spectrometer (Thermo Fischer Scientific, Waltham, MA, USA) in a data-dependent acquisition mode (DDA). Full-scan MS spectra were acquired in the Orbitrap (Scan range 350–1500 *m*/*z*, resolution 70,000; AGC target, 3 × 10^6^, maximum injection time, 100 ms). The 10 most intense peaks were selected for higher-energy collision dissociation (HCD) fragmentation at 30% of normalized collision energy. HCD spectra were acquired in the Orbitrap at resolution 17,500, AGC target 5 × 10^4^ maximum injection time 120 ms with fixed mass at 180 *m*/*z*. Charge exclusion was selected for unassigned and 1+ ions. The dynamic exclusion was set to 20 s. For CAPTURE pulldown protein identification, MS/MS spectra were searched using MaxQuant (version 1.6.3.4) and filtered in Perseus. Search parameters included trypsin or LysC cleavage with up to two missed cleavage events. Searches also permitted variable modifications of methionine oxidation or acetylation, and carbamidomethylation as fixed modification. Precursor ion tolerance was 20 ppm. Peptide assignments were filtered to a false discovery rate (FDR) of 1% on the peptide level. The data are displayed in Tables S1 and S2. For dCas9 protein identification, tandem mass spectra were searched using SEQUEST HT within Proteome discoverer PD1.4 (Thermo Fischer Scientific, version 1.4.0.288) against a database containing 284 protein entries combining dCas9 protein sequence from *Streptococcus pyogenes* and common contaminants. During database searches, cysteines (C) were considered to be fully carbamidomethylated (+57.0215, statically added), methionine (M) to be fully oxidised (+15.9949, dynamically added), all N-terminal residues to be acetylated (+42.0106, dynamically added). Two missed cleavages were permitted. Peptide mass tolerance was set at 50 ppm and 0.02 Da on the precursor and fragment ions respectively. Protein identification was filtered at FDR below 1%. The data are displayed in Table S3.

### 2.5. Western Blotting

Western blot analysis was performed as previously described [28] using approximately 10 µg of proteins from cells resuspended in Laemmli buffer (50 mM Tris pH6.8, 2% sodium dodecyl sulphate, 5% β-mercaptoethanol, 10% glycerol, 0.1% Bromophenol Blue), treated with Benzonase for 10 min at room temperature, and boiled for 3 min before loading. Blots were imaged using either X-ray film or an iBright FL1500. The antibodies used are listed in Table 2.

### 2.6. Chromatin Immunoprecipitation (ChIP)

ChIP and qPCR were performed essentially as described by [43] using approximately 1 × 10^7^ HEK293 cells crosslinked with 1% formaldehyde at room temperature for 10 min. For qPCR, the Ct value (copies/µL) for each sample was quantified relative to the standard and the IgG control Ct value was subtracted. The resulting values are the % of input being pulled down as shown in the ChIP graphs. Experiments were replicated at least three times and each ChIP sample was measured in triplicate by qPCR. Data presented represent the mean ± SEM (standard error of the mean) of three independent experiments. IgG IP was carried out for each experiment and any signal below that of IgG was considered as background and subtracted from the specific antibody value. The value for the U2 PSE primers was normalized to 1 before calculating the SEM for the other primer pairs. The antibodies used are listed in Table 2. The specificity of immunoprecipitation by SPT6, CDK12, and Cyclin K antibodies was validated by the manufacturers (SPT6 https://www.cellsignal.co.uk/products/primary-antibodies/spt6-d6j9h-rabbit-mab/15616) (accessed on 1 March 2022) (CDK12 Novus-https://www.novusbio.com/products/crkrs-antibody_nb100-87011) (accessed on 1 March 2022) (Cyclin K https://www.fortislife.com/products/primary-antibodies/rabbit-anti-cyclin-k-antibody/BETHYL-A301-939) (accessed on 1 March 2022).

The primers used for qPCR are listed in Table 1.

### 2.7. ChIP-Seq

CDK12 ChIP-seq in HEK293 CDK12as cells was performed with the LSBio CDK12 antibody listed in Table 1 following the protocol described in [43]. The CDK12as ChIP-seq data have been deposited to the GEO under the accession number GSE197372.

### 2.8. Bioinformatics Analysis

The Gencode V35 annotation, based on the hg38 version of the human genome, was used to extract the list of snRNA genes, excluding snRNA pseudogenes and pol III transcribed snRNA genes. The DNA sequence of the human *RNU2* locus (U2 snRNA genes) was obtained from GenBank (U57614.1) and re-analysed as previously described [49]. HEK293 total pol II and SPT6 ChIP-seq were obtained from GSE115290 [43]. HeLa Chromatin RNA-seq and INTS3 ChIP-seq were obtained from GSE110028 [42].

### 2.9. ChIP-Seq Data Processing

Adapters were trimmed with Cutadapt [50] version 1.18 in paired-end mode with the following options: --minimum-length 10 -q 15,10 -j 16—A GATCGTCGGACTGTAGAACTCTGAAC—a AGATCGGAAGAGCACACGTCTGAACTCCAGTCAC. Trimmed reads were mapped to the human *RNU2* gene or to the GRCh38.p13 reference sequence with STAR version 2.7.3a [51] and the parameters: --runThreadN 16 --readFilesCommand gunzip -c -k --limitBAMsortRAM 20000000000 --outSAMtype BAM SortedByCoordinate. SAMtools [52] version 1.9 was used to retain the properly paired and mapped reads (-f 3) and to remove PCR duplicates. Reads mapping to the DAC Exclusion List Regions (accession: ENCSR636HFF) were removed with BEDtools [53] version 2.29.2. SAMtools was used to obtain the number of reads mapping to the *RNU2* gene and to the human genome to calculate for each sample the normalization factor. Library-size normalized bedGraph files were created with BEDtools genomecov. Profiles across the *RNU2* gene were created with GraphPad Prism 9.1 (GraphPad Software, San Diego, CA, USA)).

### 2.10. Chromatin RNA-Seq Data Processing

Chromatin RNA-seq were analysed as previously described [54]. Briefly, adapters were trimmed with Cutadapt version 1.18 in paired-end mode with the following options: --minimum-length 10 -q 15,10 -j 16—A GATCGTCGGACTGTAGAACTCTGAAC—a AGATCGGAAGAGCACACGTCTGAACTCCAGTCAC. The remaining rRNA reads were removed by mapping the trimmed reads to the rRNA genes defined in the human ribosomal DNA complete repeating unit (GenBank: U13369.1) with STAR version 2.7.3a and the parameters --runThreadN 16 --readFilesCommand gunzip -c -k --outReadsUnmapped Fastx --limitBAMsortRAM 20000000000 --outSAMtype BAM SortedByCoordinate. The unmapped reads were mapped to the human *RNU2* gene or to the GRCh38.p13 reference sequence with STAR version 2.7.3a and the parameters: --runThreadN 16 --readFilesCommand gunzip -c -k --limitBAMsortRAM 20000000000 --outSAMtype BAM SortedByCoordinate. SAMtools version 1.9 was used to retain the properly paired and mapped reads (-f 3) and to create strand-specific BAM files. SAMtools was used to obtain the number of reads mapping to the RNU2 gene and to the human genome to calculate for each sample the normalization factor. Library-size normalized bedGraph files were created with BEDtools genomecov. Profiles across the *RNU2* gene were created with GraphPad Prism 9.1.

## 3. Results

### 3.1. Designing Guides for DCas9 Targeting

**Figure 1:**
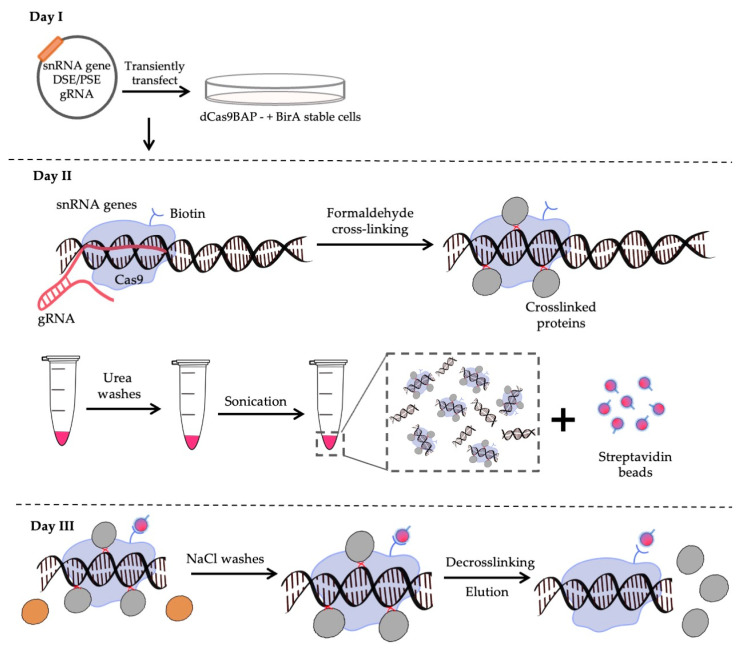
Scheme of the streptavidin–biotin CAPTURE. **Day I**: The guide (g)RNA (red line) is transiently transfected into cells stably expressing dCas9BAP (blue shape) and BirA. **Day II**: The cells are crosslinked with 2% formaldehyde to crosslink associated proteins (grey spheres), harvested, chromatin (red pellet in tube) is extracted and subjected to several 8 M urea washes. The chromatin is sonicated and incubated with streptavidin beads (red spheres with blue outline). **Day III**: The beads are washed to remove non interacting proteins (brown spheres) with 150 mM NaCl and proteins are de-crosslinked. The samples are then subjected to mass spectrometry after on-bead digestion.

CRISPR affinity purification in situ of regulatory elements (CAPTURE) exploits dCas9 directed to specific gene regions by RNA guides followed by crosslinking to capture the associated proteins [37]. As the dCas9 contains a biotin acceptor peptide (BAP), the crosslinked complexes can be purified using streptavidin. The system is encoded in three plasmids; one encoding dCas9BAP; a second encoding BirA, which biotinylates the dCas9BAP in vivo; and a third encoding the guides (Figure 1). We have used HEK293 cells as there are approximately 15 U2 6kb tandem repeats per haploid human genome [38,39] and this locus is thought to be triploid in HEK293 cells [40].

**Figure 2:**
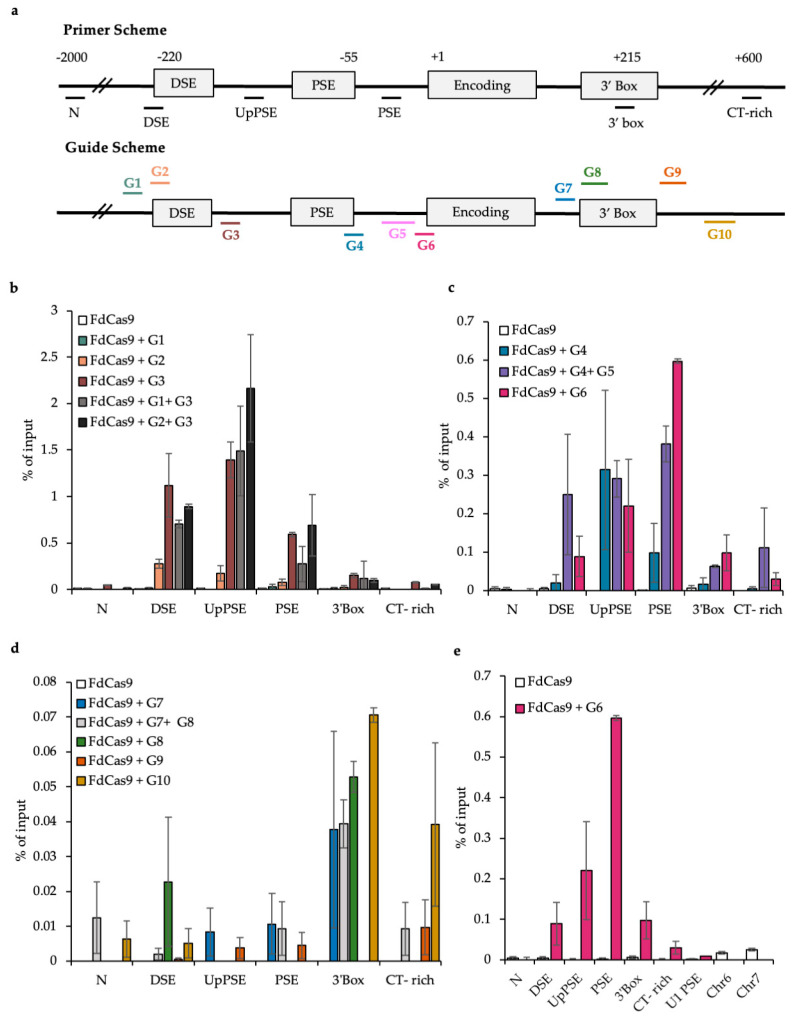
ChIP-qPCR of FLAG-tagged dCas9 (FdCas9) on the U2 snRNA gene after transient expression of guide RNAs. (**a**) Top: The position of the U2 gene qPCR primers used to amplify the ChIP products. Bottom: The position of the guide RNAs designed to target the U2 snRNA genes. N = negative control non-transcribed region. CT-rich is a CT-rich region downstream of the U2 snRNA genes. The qPCR primers and the guide RNAs are colour coded to the regions they target. (**b**) FdCas9 occupancy assessed by ChIP-qPCR on the U2 snRNA gene using an anti-FLAG antibody after transient transfection of DSE-specific guide RNAs. (**c**) PSE-specific guides. (**d**) 3′ box-specific guides. (**e**) qPCR with primers to the N, DSE, UpPSE, PSE, 3′ box, and CT-rich regions of the U2 snRNA the U1 PSE and potential off-target regions on Chr6 and Chr7 after transient transfection of G6 and anti-Flag ChIP.

We first carried out some experiments using FLAG-tagged dCas9 (FdCas9) to identify guides that are specific for the region targeted and to ensure that dCAS9 targeted to the genes does not interfere with transcription. We designed and tested a range of single and paired guides (Table 1, Figure 2a). Paired guides should result in more dCas9 recruited to the U2 snRNA genes, which could make pulldown of dCas9 easier. However, recruiting more dCas9 could interfere with factors binding to the DNA. Single guides were therefore also tested.

Guides targeting DSE, PSE, and the 3′ box were cloned into pX462 [48] with an H840A mutation in FCas9 (Materials and Methods). The plasmids were transiently transfected into HEK293 cells, and to assess whether dCas9 was being targeted to the region of interest, ChIP of the U2 snRNA genes was carried out using an antibody to the FLAG tag on FdCas9 (Figure 2). G1, G2, and G3 target the DSE (Figure 2a,b); G4, G5, and G6 target the PSE (Figure 2a,c) and G7, G8, G9, and G10 target the 3′ box (Figure 2a,d). G3 most efficiently targets FdCas9 to the DSE region; G6 most efficiently targets FdCas9 to the PSE and G10 gives the highest level of FdCas9 on the 3′ box. Interestingly, G8 may target FdCas9 to both the 3′ box and the DSE, suggesting that there is a physical link between these two regions.

ChIP-qPCR across the U2 snRNA genes shows clearly that dCas9 is not targeted to the different genomic regions with the same efficiency. For example, the recruitment of Cas9 to the 3′ box region is low relative to the DSE and PSE. This may be due to the transcriptional complex associated with this region of the U2 snRNA genes blocking FdCas9 access. In addition, pairs of guides were not more efficient than single guides. Based on these results, guides G3 and G6 were chosen for further experiments targeting the DSE and PSE regions of the U2 snRNA gene, respectively, and 3′ box region CAPTURE was not attempted.

In order to assess the specificity of the guides, the PSE region of the U1 snRNA genes was also analysed by qPCR when targeting the U2 PSE region with the G6 guide (Figure 2e). Although the PSE region is conserved between snRNA genes, G6 is specific for the U2 PSE snRNA gene region as very little FdCas9 is detected on the U1 PSE with this guide. Additionally, the sequence of the G6 guide was blasted against the whole genome and the top two potential off-target regions were analysed by qPCR. The identity between the guide and the off-target regions was 15 on chromosomes 6 and 16 on chromosome 7. However, qPCR indicates that dCas9 is not enriched on these regions when the guide is present, emphasizing that this guide is specific to the region it was designed to target. As potential Guide 3 off-target identities were lower than for Guide 6 genome-wide, we assume that this guide is also specific to the targeted region.

### 3.2. Testing the CAPTURE System

**Figure 3:**
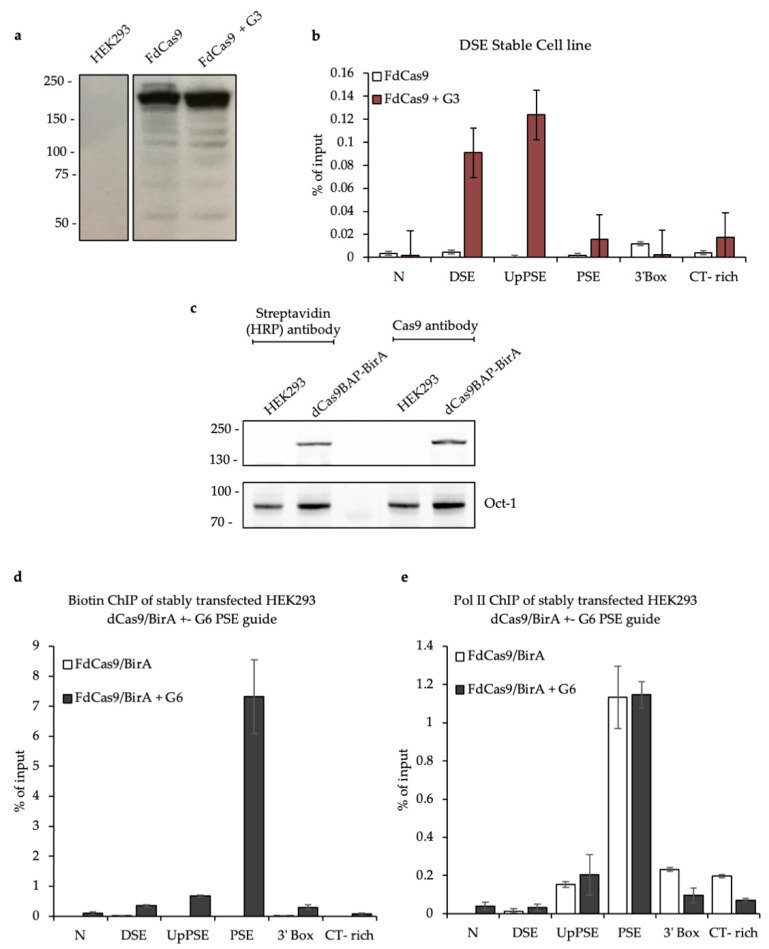
Stable expression of FdCas9 or dCas9BAP and U2 snRNA gene targeting. (**a**) Western blot of cell lines expressing FdCas9 or FdCas9 +DSE Guide 3 with an anti-Flag antibody. (**b**) ChIP-qPCR of FdCas9, using an anti-Flag antibody, across the U2 snRNA genes for both stable cell lines. (**c**) Western blot of the cell line expressing dCas9BAP and BirA with the antibodies noted above. Oct-1 serves as an internal loading control. (**d**) ChIP-qPCR of dCas9BAP across the U2 snRNA genes in dCas9BAP/BirA stable cell lines, with or without transfection of G6, using a streptavidin HRP antibody. (**e**) ChIP-qPCR of pol II across the U2 snRNA genes in dCas9BAP/BirA stable cell lines, with or without transfection of G6, using anti-pol II antibody as noted.

Once the guides were chosen, stable cell (SC) lines were generated for FdCas9 only and FdCas9 + Guide 3 (FdCas9 + G3). dCas9 expression was confirmed by Western blot (Figure 3a) and FdCas9 targeting to the U2 snRNA gene was analysed by ChIP-qPCR (Figure 3b). The results confirm that dCas9 targets the U2 DSE in the stable cell line well only when G3 is also present.

As the streptavidin–biotin interaction is stronger than an antibody–antigen interaction, stable cell lines expressing dCas9BAP and BirA were generated. Western blot confirmed that dCas9BAP is expressed and biotinylated in vivo (Figure 3c). ChIP qPCR confirmed that biotinylated dCas9 is enriched on the PSE of the U2 snRNA genes after ectopic expression of Guide 6 (Figure 3d). In addition, the presence of biotinylated dCas9BAP on the U2 PSE does not impair pol II recruitment as the pol II profile for the U2 snRNA gene measured by ChIP-qPCR is not affected when Guide 6 is present (Figure 3e).

### 3.3. CAPTURE on the U2 snRNA Gene PSE and DSE

**Figure 4:**
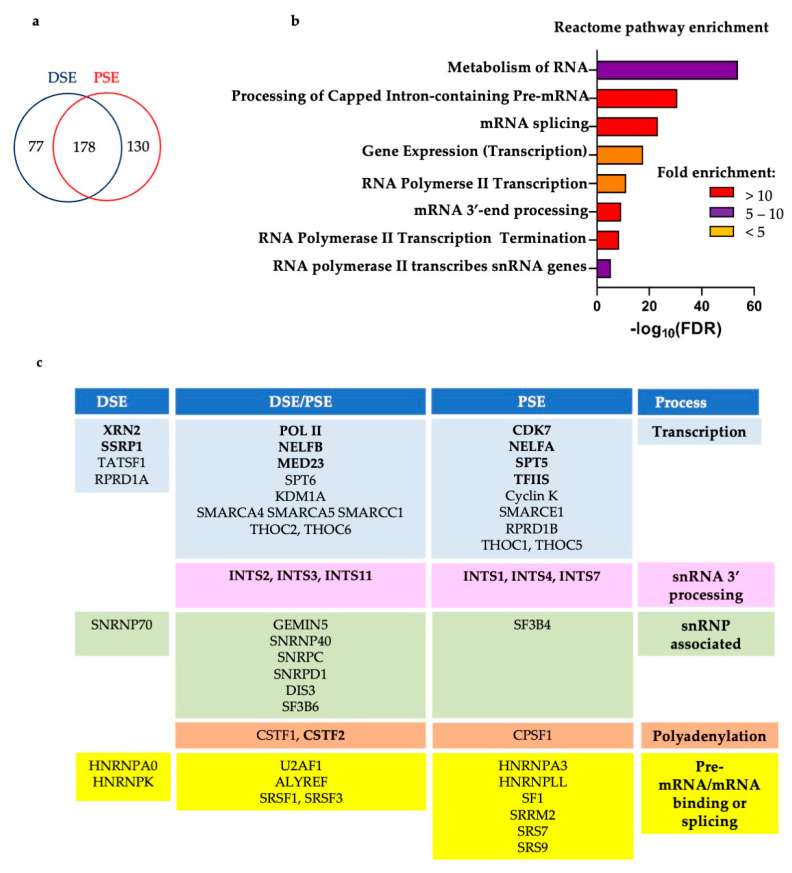
Reactome pathways and summary of the proteins CAPTUREd by the U2 snRNA gene DSE and PSE guides. (**a**) The enriched proteins detected with pull-down of DSE or PSE guides, indicating the overlap. (**b**) The list of proteins for the CAPTURE with the DSE and the PSE guides was submitted to The Gene Ontology Resource (http://geneontology.org (accessed on 1 March 2022)) for Reactome pathways enrichment analysis. (**c**) Table of selected factors enriched by CAPTURE colour-coded into the groups noted under Process on the right. On the left, factors present only in the DSE CAPTURE; on the right, factors present only in the PSE CAPTURE; in the middle, factors present both in the DSE and PSE. Cut off: 4-fold enrichment. See Tables S1 and S2 for full lists.

U2 snRNA gene CAPTURE was carried out by transiently transfecting Guides 3 or 6 into the HEK293 cell line stably expressing dCas9BAP and BirA. The cells were crosslinked with formaldehyde and pull-down of the dCas9 using streptavidin beads was carried out as detailed in Figure 1 and [36]. Crosslinking before purification of dCas9BAP and associated proteins stabilizes interactions between proteins and nucleic acids. Stringent washes with urea and NaCl enriches for chromatin-bound proteins and increases the specificity of the pull-down. Pull-down of the biotinylated dCas9BAP without any ectopically expressed guide serves as a negative control. After optimization of the ratio between the chromatin input and the number of beads used for pulldown (Materials and Methods), the mass spectrometry list obtained for the DSE region using streptavidin–biotin CAPTURE comprises 77 proteins enriched more than 4 fold when G3 is co-expressed, 130 proteins enriched more than 4 fold when G6 is co-expressed and 178 proteins enriched more than 4-fold when either guide is present (Figure 4a, Tables S1 and S2). Pull-down of dCas9 was similar for all samples (Table S3). The list of proteins from CAPTURE with the DSE and the PSE guides was submitted to The Gene Ontology Resource (http://geneontology.org (accessed on 1 March 2022)) for Reactome pathways enrichment analysis [55,56] and pathways related to transcription of snRNA genes and RNA processing were enriched (Figure 4b). Figure 4c depicts enriched proteins known or likely to be involved in transcription of snRNA genes or processing of their transcripts. Proteins known to bind to snRNAs, including U2 snRNP factors, polyadenylation factors, and several of the enriched factors that are involved in splicing or bind to pre-mRNA/mRNA are also noted. The factors already shown to be associated with snRNA genes or involved in their expression are shown in bold. Notably, Integrator subunits INTS1, INTS2, INTS3, INTS4, INTS7, and INTS11; pol II subunit RPB2; Spt5; CDK7; XRN2; MED23; NELFA; and NELFB are enriched. These have all previously been shown to play roles in expression of snRNA genes, including XRN2, which is associated with several human snRNA genes and whose knockdown can cause a termination defect [57]. TCEA1/2 (TFIIS), facilitates cleavage of the 3′ end of the nascent transcript in the pol II active site to allow backtracking if pol II stalls [58]. As it is considered a general pol II transcription factor, it may be expected to function in transcription of snRNA genes. In support of this, TFIIS has been shown to ChIP to the U2 snRNA genes [26]. The role of FACT (SSRP1) [59] in expression of snRNA genes is not clear.

Other transcription factors that were enriched include the elongation factor SPT6 [41,42]; Cyclin K, the binding partner of CDK12 and CDK13 [44]; TATSF1, which interacts with the U2 snRNP [60]; RPRD1A and RPDR1B, which are scaffolds for the recruitment of the RPAP2 CTD Ser5P phosphatase [61]; SWI/SNF chromatin-remodelling complex-associated SMARC factors [46]; and subunits of the THO elongation complex, which helps link transcription to RNA processing and export [62]. All of these factors are involved in expression of protein-coding genes but could conceivably also have roles in snRNA gene expression. Notably, SPT6 is implicated in the recruitment of Integrator to long non-coding genes to allows proper transcription termination to occur [42]. As SMARC factors are involved in chromatin remodelling [46], they may be involved in landscaping the chromatin of U2 snRNA genes. Furthermore, CDK12 and/or CDK13 may function alongside CDK7 and CDK9 to regulate these genes.

Interestingly, the protein-coding gene factors involved in termination and RNA 3′ end formation, CSTF1, CSTF2, and CPSF1 were also enriched in the CAPTURE dataset. CSTF2 (CSTF64), Pcf11, and Ssu72 have already been shown to be associated with the U2 gene by ChIP and PCF11 and Ssu72 aid termination of transcription [47].

Several subunits of snRNPs, including the SF3B4 and SF3B6 subunits of the U2 snRNP, and several pre-mRNA/mRNA binding or splicing factors, including U2AF1, SF1, SRSF1, SRSF3, SRS7, and SRS9 were also, surprisingly, enriched by CAPTURE. As U snRNA genes are intronless, these factors would have a splicing-independent role, if any, in the regulation of expression of the U2 snRNA genes. Many of these could well be interacting with the dCas9 or the associated RNA non-specifically. However, the U2 snRNP has been shown to facilitate 3′ end formation of the intronless and non-polyadenylated transcripts from replication-activated histone genes [63].

The streptavidin–biotin CAPTURE therefore appears to be quite efficient as many expected proteins were enriched, many proteins were enriched in both the DSE and PSE CAPTURE datasets and in most cases, proteins were not present at all in the negative control (Table S1).

### 3.4. SPT6 Helps to Recruit Integrator to the Human snRNA Genes

**Figure 5:**
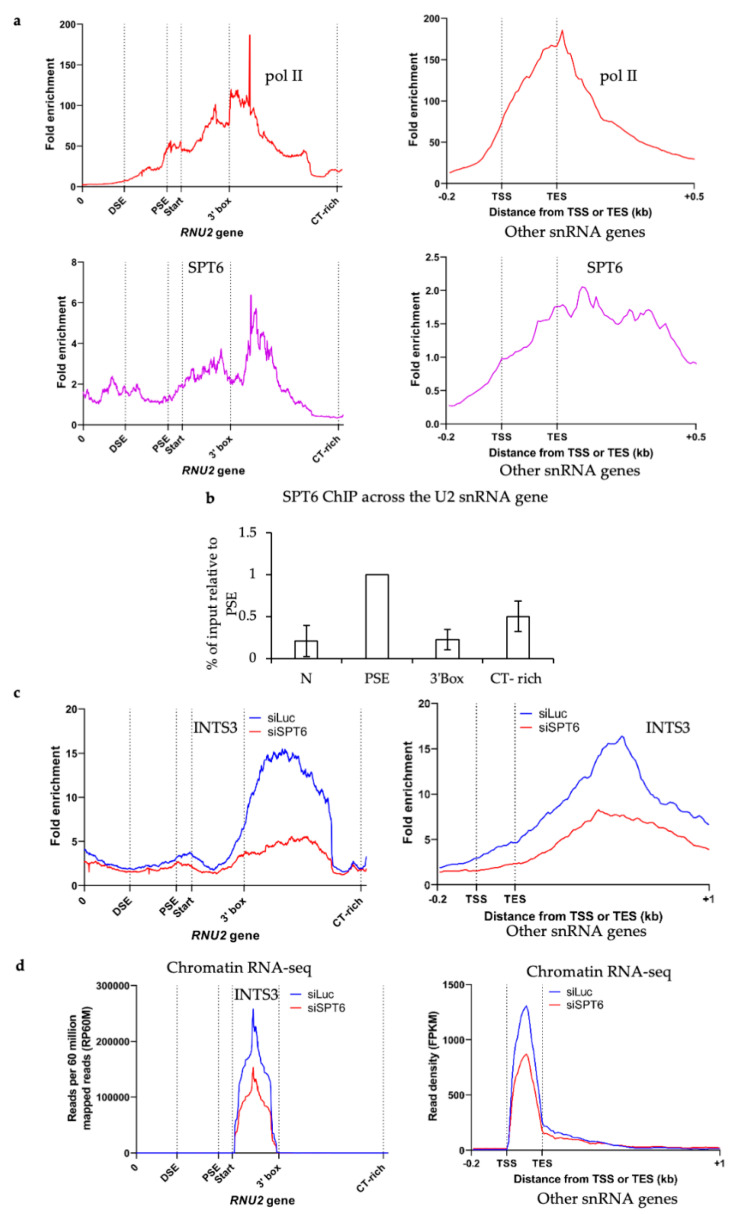
SPT6 helps to recruit Integrator to the human snRNA genes. (**a**) Re-analysis of previously published ChIP-seq of pol II (left) and SPT6 (right) over the U2 snRNA genes (RNU2) and other snRNA genes in 293 CDK12as cells [43]. (**b**) ChIP-qPCR of SPT6 across the U2 snRNA genes in 293 cells. The PSE value was normalised to 1. (**c**) Re-analysis of previously published ChIP-seq of INTS3 across the U2 snRNA genes and other snRNA genes in HeLa cells with and without siRNA-mediated knockdown of SPT6 [42]. (**d**) Re-analysis of previously published chromatin RNA-seq over the U2 snRNA genes and other snRNA genes in HeLa cells with and without siRNA-mediated knockdown of SPT6 [41].

In order to validate the association of some CAPTUREd factors with the U2 snRNA genes, we have reanalysed our previously published SPT6 ChIP-seq datasets from 293 CDK12 analogue-sensitive (as) cells (in the absence of inhibitor) [43] and found that SPT6 is associated with the transcribed region of U2 snRNA genes (RNU2 gene) and other pol II-transcribed snRNA genes (Figure 5a). SPT6 was also detected on the U2 snRNA genes in wild-type 293 cells by ChIP-qPCR (Figure 5b). In addition, knockdown of SPT6 causes loss of the INTS3 subunit of Integrator from the U2 snRNA genes and other pol II-transcribed snRNA genes and reduction in RNA from these genes as measured by chromatin RNA-seq (Figure 5c,d), indicating that SPT6 has a functional role in expression of snRNA genes.

### 3.5. CDK12 Is Associated with the U2 snRNA Genes

**Figure 6:**
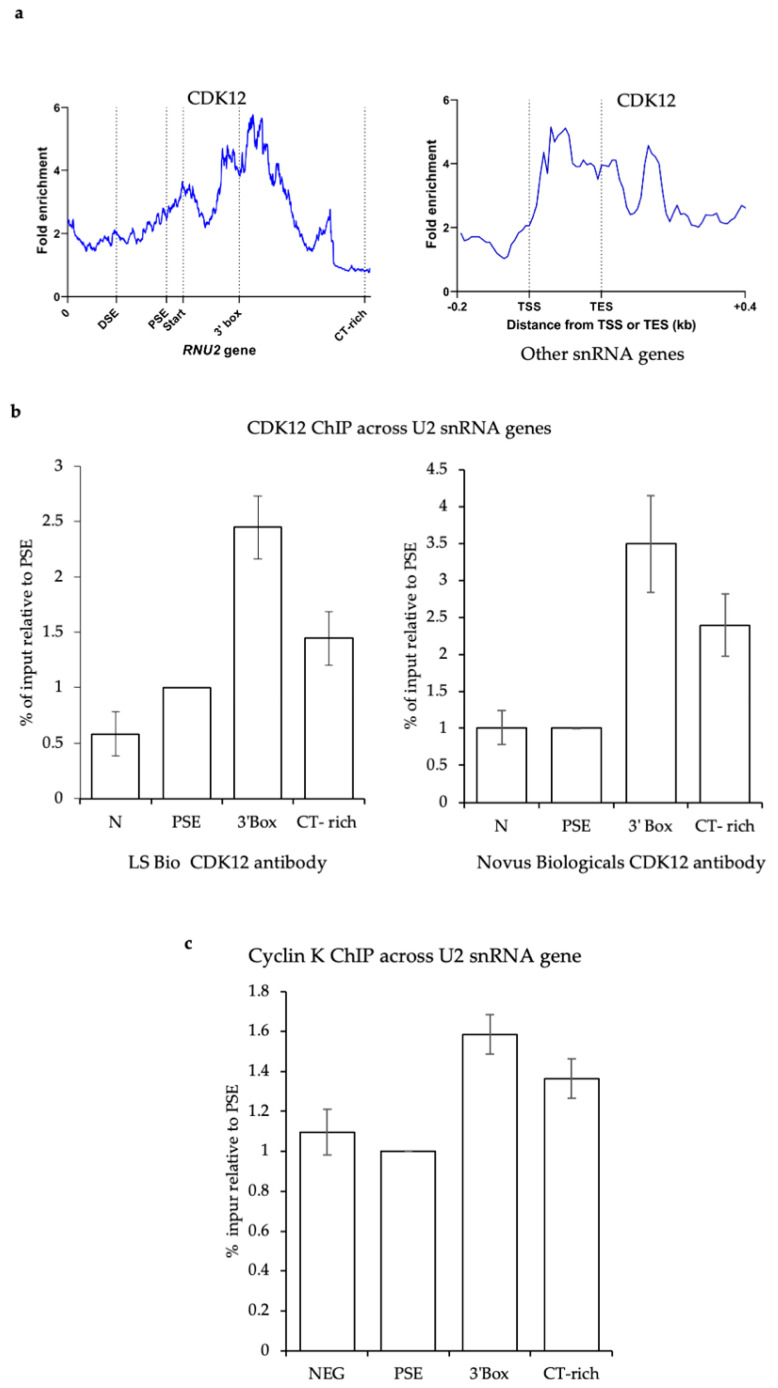
CDK12 is associated with the U2 snRNA genes. (**a**) ChIP-seq of CDK12 over the U2 snRNA genes (RNU2, left) and other snRNA genes (right) in 293 CDK12as cells [42]. (**b**) ChIP of CDK12 on the U2 snRNA genes using anti-CDK12 antibodies from LS Bio or Novus. The PSE value was normalised to 1. (**c**) ChIP of Cyclin K on the U2 snRNA genes. The PSE value was normalised to 1.

Cyclin K was enriched with the U2 PSE guide CAPTURE, suggesting that CDK12 and/or CDK13 are recruited to U2 genes. We performed CDK12 ChIP-seq in 293 CDK12as cells in the absence of inhibitor [43] and ChIP-qPCR with two different anti-CDK12 antibodies in 293 cells, indicating that this kinase is associated with the U2 snRNA genes (Figure 6a,b).

## 4. Discussion

Transcription initiation of snRNA genes is mediated by transcription factors like Oct-1, which recognize the DSE element, and by PTF binding to the PSE, which nucleates a pre-initiation complex similar to that on protein-coding genes [2,3,4,5,9,10,11,12,13,14,15,64]. However, subsequent elongation requires the snRNA gene-specific little elongation complex (LEC) [21] and finally, 3′ end formation and termination require recognition of the 3′ box RNA processing element and cleavage of the nascent RNA by the Integrator Complex [6]. It has been shown that there is compulsory coupling between the promoter element PSE and the 3′ box; transcription by pol II must initiate from a PSE-containing promoter for the 3′ box to be recognised [35,36]. Thus, sophisticated mechanisms are operating in the transcription of snRNA genes. However, these mechanisms are not yet completely understood. We carried out CAPTURE on the repeated human U2 snRNA genes as an unbiased way to further characterize the transcription machinery present on these genes.

Gratifyingly, many factors with known roles in snRNA gene expression or that had previously been shown to be associated with these genes were enriched by CAPTURE. The RPB2 subunit of pol II, CDK7, SPT5, NELF, TFIIS, XRN2, MED23, and the SSRP1 subunit of FACT and the polyadenylation factor, CSTF2 were all enriched, in addition to several subunits of Integrator, including INTS11, the catalytic subunit.

The enrichment of Cyclin K suggests that its partners CDK12 and/or CDK13 are also part of the transcription machinery and we have validated CDK12 association by ChIP and ChIP-seq. CDK12 is a transcription elongation and pol II CTD kinase [43] with many potential additional targets [65]. The pol II CTD comprises 52 repeats of the consensus heptapeptide, Y_1_S_2_P_3_T_4_S_5_P_6_S_7_. Phosphorylation of the CTD on Ser2 by CDK9 and Ser7 by CDK7 helps to recruit Integrator [2,31]. CDK12 could therefore play roles in elongation or 3′ box recognition.

We also validated the association of SPT6, which we have shown plays an important role in Integrator recruitment to the U2 snRNA genes in addition to other ncRNA genes [42]. Loss of SPT6 is therefore likely to cause a 3′ RNA processing defect in addition to affecting RNA production.

**Figure 7:**
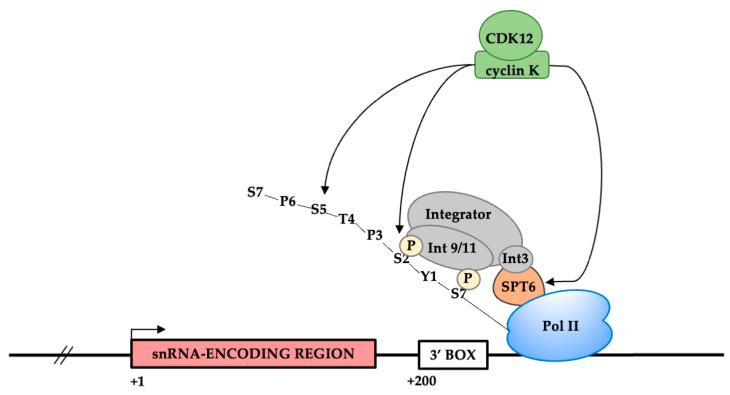
Model for the role of SPT6 and CDK12 in the expression of snRNA genes. CDK12 may phosphorylate the CTD and/or Spt6 to help recruitment of Integrator to snRNA genes or stabilization of the complex.

It will be interesting to investigate the potential roles of SPT6 and CDK12 in coupling transcription initiation from an snRNA promoter to 3′ box recognition. For example, phosphorylation of the pol II CTD and/or SPT6 by CDK12 [66] could ensure efficient recruitment of Integrator or stabilisation of this complex on snRNA genes (Figure 7).

The SWI/SNF-related, matrix-associated, actin-dependent regulators of chromatin (SMARC)A4, A5, CC1, and CE1 factors are part of the SWI/SNF chromatin remodelling complex [46]. The SWI/SNF complex can remodel chromatin and relocate nucleosomes to facilitate transcription and could be involved in regulating transcription of snRNA genes by changing the chromatin environment of the PSE region, allowing transcription factors to bind and promote transcription. PTF binding to the PSE is required to maintain an open chromatin structure as the nucleosome density across the U1 and U2 snRNA genes increases after PTF knockdown [47]. SMARC factors could therefore be recruited by PTF to help remove nucleosomes from the transcribed region. In addition, a nucleosome between the DSE and PSE of snRNA genes is important to bring these two promoter elements close together [67,68,69,70] and SWI/SNF could play a role in positioning this nucleosome. FACT may collaborate with SPT6 to ensure efficient elongation. The roles of TATSF1, RPRD1A/B, and the THO complex in snRNA gene expression would also be interesting to explore.

CAPTURE has therefore implicated more ‘protein-coding gene’ transcription and RNA processing factors in snRNA gene expression.

In common with other published dCas9 pull-down studies, including Cas9 locus-associated proteome (CLASP) of *Drosophila melanogaster* histone genes [71], we retrieved many RNA-associated proteins, including snRNA-associated factors and splicing factors but no classical sequence-specific DNA-binding factors. The DNA-binding factors Oct-1 and PTF/SNAPc/PBP are conspicuous by their absence. We have no clear explanation of why this should be if the promoter region of the snRNA genes is being pulled down as expected. The guide RNAs used and the associated Cas9 may interfere with the stable binding of factors to the DNA template. However, we see no loss of pol II from the genes, as we would expect if promoter factors were affected. Alternatively, the RNA associated with the Cas9 may favour the enrichment of factors crosslinked to RNA, some of which may be non-specific. These will increase the complexity of the sample and could crowd out DNA-binding factors. Validation of the specific association of these RNA-binding factors is therefore necessary, although they are enriched when dCas9 is directed to chromatin by a guide.

Despite this, CAPTURE has helped us to identify SPT6 and CDK12/Cyclin K as novel components of the transcription machinery associated with the human U2 snRNA genes. Thus, although the association and potential role of interesting chromatin remodelling, transcription, and RNA processing factors—picked up by CAPTURE on these genes—remain to be validated, streptavidin–biotin CAPTURE has proven useful to identify factors involved in expression of the human U2 snRNA gene.

## Figures and Tables

**Table 1:** Sequence of primers for dCas9 cloning and sequencing, U2 snRNA guides, and U2 snRNA qPCR primers. All primers are shown 5′ to 3′.

Name	Forward Primer	Reverse Primer
dCas9 EcoVR	GAATGATATCGTGCTGACCCTGACAC	GTCCACATCGTAGTCGGACAG
dCas9 FseI	GCCATCGTGCCTCAGAGCTTTC	GAACGGCCGGCCTTTTTCGTGG
dCas9 ExF Seq	GGGCACATACCACGATCTGCTG	
dCas9 Mut Seq	GGCAAGAGCGACAACGTGCCCTC	
dCas9 ExR Seq	GGATTCTCCTCGACGTCACCGC	
G1	CACCCCGCCCTTTCACAGAGGGCG	AAACCGCCCTCTGTGAAAGGGCGG
G2	CACCATGCCCCGCCCTTTCACAGA	AAACTCTGTGAAAGGGCGGGGCAT
G3	CACCAAATGAAAGCCCGGGAACGC	AAACGCGTTCCCGGGCTTTCATTT
G4	CACCTCTCATCCACATTCAAGTCG	CACCTCTCATCCACATTCAAGTCG
G5	CACCGACGGTGACGGCGGGCGCGA	AAACTCGCGCCCGCCGTCACCGTC
G6	CACCAAGGCGAGCGCATCGCTTCT	CACCAAGGCGAGCGCATCGCTTCT
G7	CACCCCGTCTACCGCCCGCACATC	AAACGATGTGCGGGCGGTAGACGG
G8	CACCGCACGCTGTCGTTTCCACCG	CACCGCACGCTGTCGTTTCCACCG
G9	CACCCCGGAAGGGTTTCGCGTCAT	CACCCCGGAAGGGTTTCGCGTCAT
G10	CACCAGTGGGTGGCGACCTTTTAA	CACCAGTGGGTGGCGACCTTTTAA
U2 N	GGAGCGGAGCGTTCTCTGTCTCCCC	AGAGTGTGAGCCCTCATTCACGCCC
U2 DSE	TGGCTCGATACGAACAAGGAAG	GTTCCCGGGCTTTCATTTCG
U2 UpPSE	GGGAACGCCGAAGAAGCACGGG	CCCCAGCCTCGCTCCTTGCCC
U2 PSE	ATGAGAGTGGGACGGTGA	CACTTGATCTTAGCCAAAAGG
U2 3′ box	ACGAGTCCTGTGACGCGCCGGCTTG	CTCCGGGTGGGTCCCATTCCTTTAA
U2 CT-rich	CCTCCCCGCCTCTCCCTCGCTC	GGACAAATAGCCAACGCATGCGG
U1 PSE	GGAAAGGGCTCGGGAGTGCGCG	CAGGTAAGTATGAGAGCTTGGGC
Chr6 off target	AGACTACACGATACAACATCCAC	AGCAGGAATCAGAACTCCCATC
Chr7 off target	AGCAGGAATCAGAACTCCCATC	ACCAAGGAGGAAAGGTAGTAGC

**Table 2:** List of antibodies used for ChIP or Western blot.

Antibodies	Reference
AffiniPure Donkey Anti-Rabbit IgG (H + L)	Jackson ImmunoResearch 711-005-152
Anti-CRISPR-Cas9	Abcam ab191468
CDK12	LS Bio LS-C288466-100
CDK12	Novus Biologicals NB100-87011
Cyclin K	Bethyl Laboratories A301-939A
Oct-1	Bethyl Laboratories A301-717A
RNA Polymerase II	Novus Biologicals NBP2-32080
SPT6	Cell Signalling 15616
Streptavidin (HRP)	Abcam ab7403

## Data Availability

The DNA sequence of the human *RNU2* locus (U2 snRNA genes) was obtained from GenBank (U57614.1) and re-analyzed as previously described [49]. HEK293 total pol II and SPT6 ChIP-seq were obtained from GSE115290 [42]. HeLa Chromatin RNA-seq and INTS3 ChIP-seq were obtained from GSE110028 [41]. CDK12 ChIP-seq is available at GSE197372. Mass spectrometry-proteomics datasets were submitted to ProteomeXchange via the Pride database (PXD033580).

## Supplementary Materials

The following supporting information can be downloaded at: https://www.mdpi.com/article/10.3390/biom12050704/s1, Table S1: Proteomic database search results; Table S2: List of enriched proteins for DSE and PSE; Table S3: dCas9 mass spectrometry protein identification in CAPTURE dCas9 alone, DSE and PSE samples.
